# Plasma extracellular vesicle long RNA profiling identifies a predictive signature for immunochemotherapy efficacy in lung squamous cell carcinoma

**DOI:** 10.3389/fimmu.2024.1421604

**Published:** 2024-08-05

**Authors:** Xin Zhang, Jiatao Liao, Wenyue Yang, Qiaojuan Li, Zhen Wang, Hui Yu, Xianghua Wu, Huijie Wang, Si Sun, Xinmin Zhao, Zhihuang Hu, Jialei Wang

**Affiliations:** ^1^ Department of Thoracic Medical Oncology, Fudan University Shanghai Cancer Center, and Shanghai Key Laboratory of Medical Epigenetics, International Co-laboratory of Medical Epigenetics and Metabolism, Institutes of Biomedical Sciences, Shanghai Medical College, Fudan University, Shanghai, China; ^2^ Department of Oncology, Shanghai Medical College, Fudan University, Shanghai, China; ^3^ Institute of Thoracic Oncology, Fudan University Shanghai Cancer Center, Shanghai, China

**Keywords:** extracellular vesicles, lung squamous cell carcinoma, immunochemotherapy, predictive signature, RNA sequencing

## Abstract

**Introduction:**

The introduction of Immune Checkpoint Inhibitors (ICIs) has marked a paradigm shift in treating Lung Squamous Cell Carcinoma (LUSC), emphasizing the urgent need for precise molecular biomarkers to reliably forecast therapeutic efficacy. This study aims to identify potential biomarkers for immunochemotherapy efficacy by focusing on plasma extracellular vesicle (EV)-derived long RNAs (exLRs).

**Methods:**

We enrolled 78 advanced LUSC patients undergoing first-line immunochemotherapy. Plasma samples were collected, and exLR sequencing was conducted to establish baseline profiles. A retrospective analysis was performed on 42 patients to identify differentially expressed exLRs. Further validation of the top differentially expressed exLRs was conducted using quantitative reverse transcription PCR (qRT-PCR). Univariate Cox analysis was applied to determine the prognostic significance of these exLRs. Based on these findings, we developed a predictive signature (p-Signature).

**Results:**

In the retrospective analysis of 42 patients, we identified 460 differentially expressed exLRs, with pathways related to leukocyte migration notably enriched among non-responders. Univariate Cox analysis revealed 45 exLRs with prognostic significance. The top 6 protein-coding exLRs were validated using qRT-PCR, identifying CXCL8, SSH3, and SDHAF1 as differentially expressed between responders and non-responders. The p-Signature, comprising these three exLRs, demonstrated high accuracy in distinguishing responders from non-responders, with an Area Under the Curve (AUC) of 0.904 in the retrospective cohort and 0.812 in the prospective cohort.

**Discussion:**

This study highlighted the potential of plasma exLR profiles in predicting LUSC treatment efficacy. Intriguingly, lower p-Signature scores were associated with increased abundance of activated CD4+ and CD8+ T cells, indicating a more robust immune environment. These findings suggest that the p-Signature could serve as a valuable tool in guiding personalized and effective therapeutic strategies for LUSC.

## 1 Introduction

Lung cancer is acknowledged as the leading cause of cancer-related deaths worldwide, consistently ranking as the most prevalent cancer (1). Within the spectrum of non-small cell lung cancer (NSCLC), lung squamous cell carcinoma (LUSC) represents approximately 20% to 30% of cases, making it the second most common subtype of NSCLC (2). The therapeutic options for advanced and metastatic LUSC have traditionally been constrained to chemotherapy, largely due to the lack of actionable genetic aberrations. The recent advent of immune checkpoint inhibitors (ICI), particularly those targeting the programmed cell death 1 (PD-1) pathway, has revolutionized LUSC treatment (3, 4). PD-1 inhibitors have shown notable clinical success, both as monotherapy in patients with a PD-1 tumor proportion score (TPS) ≥50% and as part of combination therapies with chemotherapy, irrespective of PD-1 expression levels (5–7).

The introduction of immunotherapy has opened new avenues of hope for lung cancer patients, yet identifying those who will most benefit remains a significant challenge. Currently, only about 20% of patients with advanced-stage NSCLC derive considerable benefits from immunotherapy (7). Biomarkers such as PD-L1 expression, tumor mutation burden (TMB), and microsatellite instability (MSI) or mismatch repair deficiency (dMMR) are associated with clinical efficacy (8–11). However, these markers are predominantly validated in the context of ICI monotherapy, and their predictive value in the setting of immunochemotherapy, especially when PD-L1 expression and TMB levels vary, is less clear (6). Furthermore, the utility of MSI/dMMR in lung cancer is somewhat limited due to its rare occurrence, less than 1% (12). Therefore, further research and exploration of better biomarkers are imperative to tailor immunochemotherapy.

Extracellular vesicles (EVs), secreted by a variety of cell types, contain a diverse array of proteins, nucleic acids, and lipids. These components provide insights into the physiological and pathological states of the source cells (13, 14). EV-based liquid biopsy has recently been recognized as a promising method for tumor profiling, prognostication, monitoring treatment efficacy, and identifying therapeutic targets (15, 16). Significantly, studies have underscored the role of EV-derived miRNA and PD-L1 in predicting the effectiveness of immunotherapy in advanced NSCLC (17, 18). In our previous work, we have demonstrated that EV-derived long RNA (exLRs), particularly CD160, shows potential as a prognostic biomarker for lung adenocarcinoma (LUAD) (19). However, the role of exLRs in the context of immunochemotherapy for LUSC has not been thoroughly investigated.

In this study, we employed an optimized exLR sequencing technique previously developed by our team (20) to conduct a comprehensive profiling of plasma exLRs in 78 advanced LUSC patients receiving first-line immunochemotherapy. Our analysis led to the identification of a LUSC-specific predictive signature, comprising three distinct exLRs. This signature has shown significant potential in accurately predicting the efficacy of immunochemotherapy in LUSC patients, marking a notable advancement in personalized cancer treatment.

## 2 Material and methods

### 2.1 Participants and blood samples

A total of 78 patients with locally advanced or metastatic LUSC and 51 healthy individuals were enrolled in this study at Fudan University Shanghai Cancer Center (FUSCC) between January 2020 and October 2022. Patient inclusion criteria include histologically confirmed diagnosis of inoperable stage III to IV LUSC, absence of targetable EGFR/ALK/ROS1 mutations, treatment-naïve status, and a baseline Eastern Cooperative Oncology Group (ECOG) performance status score of 0 or 1. The clinical stage was determined according to the eighth edition of the American Joint Committee on Cancer (AJCC) TNM staging system (21). Tumor PD-L1 expression levels of 47 LUSC patients were measured using the Dako IHC 22C3 pharmDx assay. All patients received ICIs (pembrolizumab or tislelizumab) in combination with chemotherapy (paclitaxel and carboplatin) every three weeks for four cycles, followed by ICIs maintenance every three weeks until disease progression or unacceptable toxicity.

### 2.2 Efficacy assessment

The tumor response was assessed every two treatment cycles using the Response Evaluation Criteria in Solid Tumors (RECIST, version 1.1) (22). Patients without disease progression or death by the cutoff date, or those who were lost to follow-up before progression or death at their last contact were censored. We utilized progression-free-survival (PFS), overall survival (OS), objective response rate (ORR) to access immunochemotherapy efficacy. PFS was defined as the time from the start of immunochemotherapy until either disease progression or death from any cause. OS was defined as the time from the initiation of immunochemotherapy until death from any cause. The ORR was calculated as the proportion of patients who achieve a complete response (CR) or partial response (PR) as the best overall response. Responders were defined as patient who achieved CR or PR as the best overall response during immunochemotherapy with PFS≥6 months. Non-responders were defined as patients who achieved stable disease (SD) or progressed disease (PD) as the best overall response, or patients with PFS<6 months.

### 2.3 Isolation of EVs and EV-derived long RNA

The EVs were purified from a 1 mL plasma sample using the exoRNeasy Serum/Plasma Kit (Qiagen, Cat. No. 77144, Hilden, Germany). Briefly, thawed plasma was mixed with binding buffer and added to the exoEasy membrane affinity spin column to bind the vesicles to the membrane. For transmission electron microscopy (TEM), size distribution measurement, and Western blotting, the EVs were eluted with 400 μL of XE elution buffer (Qiagen, Cat. No. 76214). Ultrafiltration was performed at 12,000 rpm for 17 minutes to reduce the eluate volume to 50 µL, followed by buffer exchange with phosphate-buffered saline (PBS) using an Amicon Ultra-0.5 Centrifugal Filter with a 10 kDa molecular weight cutoff (Merck Millipore, Germany). For EV RNA isolation, the EVs were lysed on the column using QIAzol (Qiagen), and total RNA was subsequently eluted with 18 µL of RNase-free water. See the **Supplementary Methods** for details.

### 2.4 RNA-seq analysis

Extracellular vesicle-derived long RNA isolated from 1mL of plasma was treated with DNase I (NEB, Ipswich, Massachusetts, USA) to remove DNA. Strand-specific RNA-seq libraries were prepared using the SMARTer Stranded Total RNA-seq Kit (Clontech, Palo Alto, California, USA). Library quality was analyzed using a Qubit fluorometer (Thermo Fisher Scientific, Waltham, Massachusetts, USA) and Qsep100 (BiOptic, New Taipei City, Taiwan). ExLR-seq was performed on an Illumina sequencing platform (San Diego, California, USA) with 150 bp paired-end run metrics. The raw sequencing reads were filtered by FastQC (version 0.11.8) and aligned to the Gencode human genome (GRCh38) using the read aligner STAR (version 2.7.1a). Gene expression levels were then quantified by featureCounts (Version 1.6.3) and transformed into transcripts per kilobase million (TPM). Annotation information of mRNA, lncRNA and pseudogene genes were retrieved from the GENCODE database (Human, version 29).

### 2.5 RT-qPCR analysis

Total EV RNA isolated from 1mL of plasma were treated with DNase I (NEB, Ipswich, Massachusetts, USA) to remove DNA. Then the cDNA was synthesized using Evo M-MLV RT Master Mix (Accurate Biology, Hunan, China). Gene expression levels were measured by qRT-PCR using SYBR Premix Ex Taq (Accurate Biology, Hunan, China). The relative expression levels of exLRs were calculated using the comparative Ct method normalized to Actin. The primers for Quantitative PCR (qPCR) are listed in **Supplementary Table 1**.

### 2.6 Data and statistical analyses

The raw read counts from RNA-seq were transformed into TPM values to standardize the expression levels of exLR genes across samples. Genes with low overall frequencies (expressed in less than 30% of all analyzed samples) were removed, and the remaining exLRs were utilized for subsequent analysis. To assess the differences in exLRs expression between responders and non-responders, the limma R package was employed, utilizing fold change (FC) and comparing them with the Mann-Whitney U-test. ExLRs with FC >1.5 and false discovery rate (FDR) <0.05 were considered as statistically significant differentially expressed genes (DEGs). Gene Ontology (GO) biological process (BP) enrichment analysis and Kyoto Encyclopedia of Genes and Genomes (KEGG) pathway analysis were conducted on the DEGs to determine the pathway enrichment and significant molecular mechanisms of the different groups. The FDR was controlled using Benjamini-Hochberg adjustment.

To identify the exLRs for predicting the efficacy of LUSC immunochemotherapy, we employed a multistep approach. First, we identified the exLRs that were differentially expressed between the responders and the non-responders, considering a FDR <0.05 and FC >1.5. Next, we analyzed the exLRs associated with PFS using a Cox regression model, considering a significance level of p < 0.05. The genes that showed a significant correlation with PFS were considered as survival-related genes. Subsequently, six significantly differentially expressed (FC >2) protein coding exLRs were selected to detected by real-time quantitative PCR (qRT-PCR) in both retrospective cohort and prospective cohort. Finally, three differential expression exLRs were used for the construction of a least absolute shrinkage and selection operator (LASSO)-COX regression model for efficacy prediction. We utilized the R software package glmnet to integrate progression-free survival time, survival status, and gene expression data, and performed regression analysis using the LASSO-COX method. Furthermore, we used fivefold cross-validation and the Akaike information criterion (AIC) to estimate the expected generalization error and the select optimal value of the ‘1-se’ lambda parameter to obtain the optimal model.

## 3 Results

### 3.1 Patient characteristics

In this study, a total of 78 patients diagnosed with LUSC and 51 healthy individuals were included (**Supplementary Table 2**). Within the LUSC patient group (**Table 1**), males were predominantly represented, with a significant proportion being current or former smokers. The majority of patients in both the retrospective (N=42) and prospective (N=36) cohorts were diagnosed with stage IV cancer, exhibiting common metastatic sites primarily in the chest region. The PD-L1 status was unknown for 28.58% of the retrospective cohort and 74.99% of the prospective cohorts. In the retrospective cohort 37 (88.10%) patients received pembrolizumab, while 5 (11.90%) patients received tislelizumab. In the prospective cohort 13 (36.11%) patients received pembrolizumab, and the remaining 23 (63.89%) received tislelizumab. There were no significant differences between the two cohorts regarding age, gender, stage, metastatic sites, and response to immunochemotherapy. As of the data cutoff in June 2023, the median follow-up duration was 26.5 months for the retrospective cohort and 11 months for the prospective cohort. The ORR was 66.67% in both cohorts. Specifically, 26 patients (61.90%) in the retrospective cohort and 22 patients (61.11%) in the prospective cohort were classified as responders to immunochemotherapy.

**Table 1:** LUSC patient cohort and baseline characteristics.

Characteristic	Retrospective cohort (N=42)	Prospective cohort (N=36)	P value
**Age, median (IQR)**	64 (61-69)	68 (64-72)	0.562
**Gender, n (%)**			0.650
Female	2 (4.76%)	1 (2.78%)	
Male	40 (95.24%)	35 (97.22%)	
**Smoking history, n (%)**			0.818
Never	4 (9.52%)	4 (11.11%)	
Previous or current	38 (90.48%)	32 (88.89%)	
**PD-L1 TPS, n (%)**			0.000
<1%	12 (28.57%)	2 (5.56%)	
1%~49%	13 (30.95%)	6 (16.67%)	
≥50%	5 (11.90%)	1 (2.78%)	
Undetermined	12 (28.58%)	27 (74.99%)	
**Stage, n (%)**			0.630
III	15 (35.71%)	11 (30.56%)	
IV	27 (64.29%)	25 (69.44%)	
**Metastatic site, n (%)**			
Chest	15 (35.71%)	21 (58.33%)	0.412
Adrenal gland	5 (11.90%)	3 (8.33%)	0.604
Liver	7 (16.67%)	6 (16.67%)	1.000
Brain	3 (7.14%)	1 (2.28%)	0.384
Bone	9 (21.42%)	7 (19.44%)	0.829
**PD-1 inhibitor, n (%)**			
Tislelizumab	5 (11.90%)	23 (63.89%)	0.000
Pembrolizumab	37 (88.10%)	13 (36.11%)	
**Response, n (%)**			0.943
Responder	26 (61.90%)	22 (61.11%)	
Non-responder	16 (38.10%)	14 (38.89%)	

IQR, interquartile range.

### 3.2 EV isolation and exLR-seq

**Figure 1:**
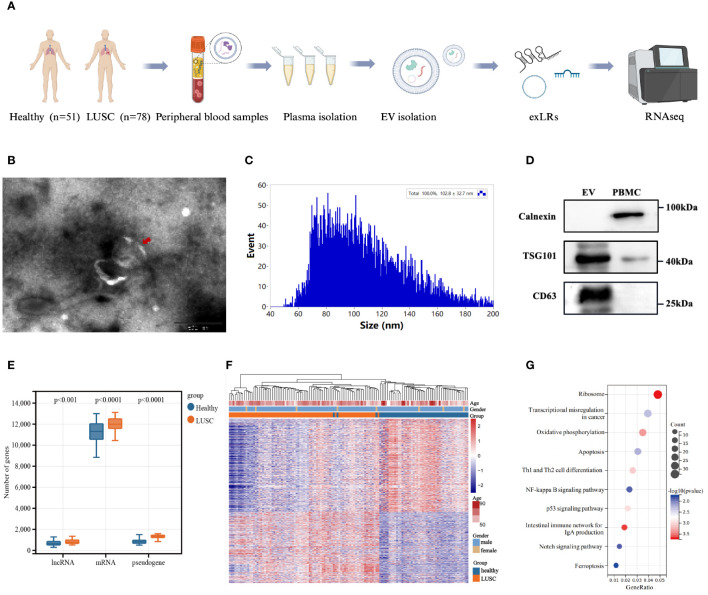
Extracellular vesicle-derived long RNA (exLR) profiles in lung squamous carcinoma (LUSC) patients and healthy individuals. **(A)** Workflow of extracellular vesicle (EV) extraction and exLR-seq. **(B)** Transmission electron microscopy image of isolated vesicles. **(C)** Particle size analysis of isolated vesicles. **(D)** Western blots of EV markers TSG101 and CD63 expression in isolated vesicles and peripheral blood mononuclear cell (PBMC). **(E)** Distribution of different exLRs per sample between LUSC patients and healthy individuals. **(F)** Heatmap of unsupervised hierarchical clustering of the differentially expressed exLRs between LUSC patients and healthy controls. Differential expression was defined by fold change >1.5 and adjusted p < 0.05. **(G)** KEGG pathway enrichment analysis for the differentially expressed exLRs.

The baseline plasma EVs were isolated from 78 LUSC patients and 51 healthy donors, and the exLR-seq technique was applied to each plasma sample (**Figure 1A**). Under transmission electron microscopy, the isolated vesicles exhibited a rounded, cup-shaped morphology with a membrane enclosure (**Figure 1B**). Through flow cytometry analysis, a diverse population of spherical nanoparticles was identified (**Figure 1C**). Western blot analysis displayed enriched expression of EV markers, TSG101 and CD63, within the isolated vesicles, contrasting with PBMCs (**Figure 1D**). These findings suggest that the isolated EVs may predominantly consist of exosomes.

Roughly 15,000 annotated genes, encompassing mRNAs, lncRNAs and pseudogenes, were consistently identified in both plasma samples from healthy donors and LUSC patients. Within the detected exLRs, a majority constituted mRNAs. Notably, the quantities of enriched mRNAs, lncRNAs, and pseudogenes were higher in LUSC patients than in healthy donors (**Figure 1E**). We identified 1,134 upregulated and 1,486 downregulated exLRs in LUSC patients compared with healthy donors (FDR <0.05, FC >1.5). Unsupervised hierarchical clustering revealed a clear separation of LUSC and healthy samples (**Figure 1F**). Furthermore, the KEGG pathway analysis illuminated the significance of differentially expressed exLRs enriched in cancer-related pathway, such as transcriptional misregulation in cancer, the NF-kappa B signaling pathway, and the p53 signaling pathway (**Figure 1G**).

### 3.3 Analysis of baseline exLRs among immunochemotherapy responders and non-responders within the retrospective LUSC patient cohort

**Figure 2:**
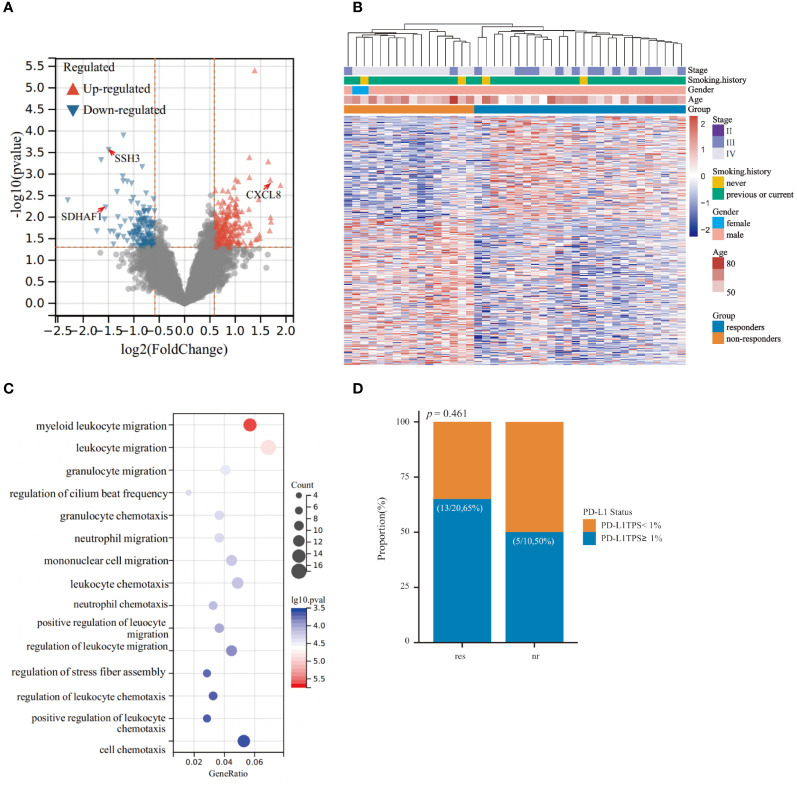
Comparison of responders and non-responders in the retrospective cohort of lung squamous cell carcinoma (LUSC) patients. **(A)** Volcano plot showing the 360 differential expressed EV-derived long RNAs (exLRs) between responders and non-responders within the retrospective cohort. Differential expression was defined by fold change >1.5 and adjusted p < 0.05. **(B)** Heatmap of unsupervised hierarchical clustering of the differentially expressed exLRs. **(C)** Top 15 significantly enriched GO-BP pathways analyzed from differentially expressed exLRs. **(D)** Histogram illustrating the distribution of PD-L1 status between responders and non-responders.

Firstly, we conducted a comparative analysis of baseline exLR expression profiles between immunochemotherapy responders (n=26) and non-responders (n=16) within the retrospective LUSC patient cohort to identify exLR-based biomarkers associated with treatment outcome. A total of 460 exLRs exhibited statistical significance (P<0.05, FC>1.5) (**Figure 2A**). Notably, unsupervised hierarchical clustering unveiled a distinct separation between responders and non-responders, underscoring the potential of exLRs as prognostic biomarkers (**Figure 2B**). Moreover, GO-BP enrichment analysis indicated the DEGs were significantly enriched in inflammation-related pathways, especially those linked to leukocyte migration and chemotaxis (**Figure 2C**). These findings suggested a pivotal role of distinct inflammatory statuses in shaping responses to immunochemotherapy among LUSC patients. To further elucidate prognostic-associated exLRs, we performed univariant Cox regression analysis to explore the correlation between baseline exLR expression levels and patients’ PFS data. Through this analysis, we successfully identified 45 exLRs with prognostic significance (**Supplementary Figure S1A**). Considering that tumor PD-L1 expression level, peripheral platelet to lymphocyte ratio (PLR), lymphocyte to monocyte ratio (LMR), neutrophil to lymphocyte ratio (NLR) and serum LDH level have been reported to correlate with ICI therapy efficacy (23–25), we proceeded to verify PD-L1 status in 30 LUSC patients, along with baseline PLR, LMR, NLR and serum LDH level in the entire cohort of 42 LUSC patients. Our results demonstrated that PD-L1 status, PLR, LMR, NLR and serum LDH level did not significantly differ between responders and non-responders (**Figure 2D**, **Supplementary Figures S1B, C**).

### 3.4 Establishment of exLRs-based predictive signature for immunochemotherapy outcomes within LUSC patients

**Figure 3:**
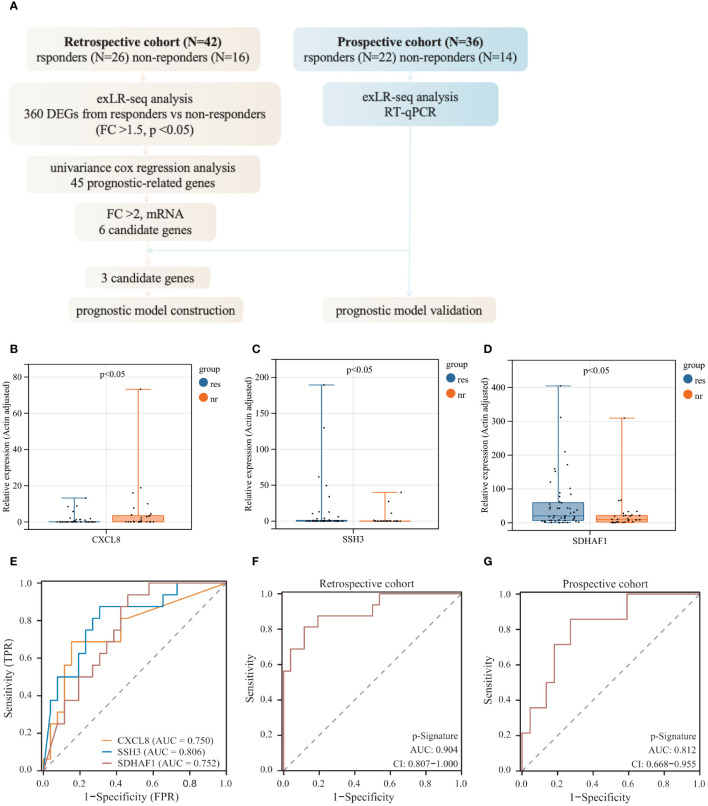
Predictive model construction for immunochemotherapy efficacy. **(A)** Workflow of prognostic model construction. **(B–D)** Relative RNA expression levels of 3 exLRs that exhibit differential expression and consistent expression trends between responders and non-responders. **(E)** ROC analysis of 3 candidate exLRs. **(F, G)** ROC analysis of the p-signature in the retrospective cohort **(F)** and the prospective cohort **(G)**.

The workflow for identifying a predictive signature for immunochemotherapy outcomes in LUSC patients was depicted in **Figure 3A**. Initially, we selected 6 significantly differentially expressed (FC>2) protein coding exLRs (CXCL8, CXCL10, RNF25, ESM1, SSH3, SDHAF1) from 45 prognostic-related exLRs as candidate biomarkers. Subsequently, we performed ROC analyses to evaluate their predictive potential. SSH3 exhibited the highest areas under the curve (AUC) of 0.806, while the other exLRs achieved AUC values ranging from 0.661 to 0.752 (**Supplementary Figure S2A**). To validate the predictive value of these exLRs in the retrospective cohort, we further enrolled 36 LUSC patients including 22 responders and 14 non-responders. The expression levels of these 6 candidate biomarkers were detected by qRT-PCR. The results indicated that the expression of 3 candidate exLRs (CXCL8, SDHAF1, SSH3) displayed significant differences and consistent expression trends with RNA-seq data between responders and non-responders (**Figures 3B–D**, **Supplementary Figures S2B–D**).

In an effort to develop a more effective predictive model based on exLRs, we tried to construct a multi-biomarker predictive model based on the three identified exLRs. Employing the LASSO-COX regression model, we established a prognostic model and generated an exLR based p-signature for LUSC patients who received immunochemotherapy (**Supplementary Figures S2E, F**). The exLR based p-Signature, comprising the three exLRs, successfully distinguished the responders from non-responders with an impressive AUC of 0.904 (95% confidence interval (CI): 0.807-1.000) in the retrospective cohort, demonstrating significantly higher accuracy than the exLR alone (**Figures 3E, F**). Subsequently, we applied the p-signature to the prospective cohort, where it detected responders with an AUC of 0.812 (95% CI: 0.668-0.955) (**Figure 3G**).

### 3.5 Performance of the exLR-derived p-signature in predicting immunochemotherapy outcomes in LUSC patients

**Figure 4:**
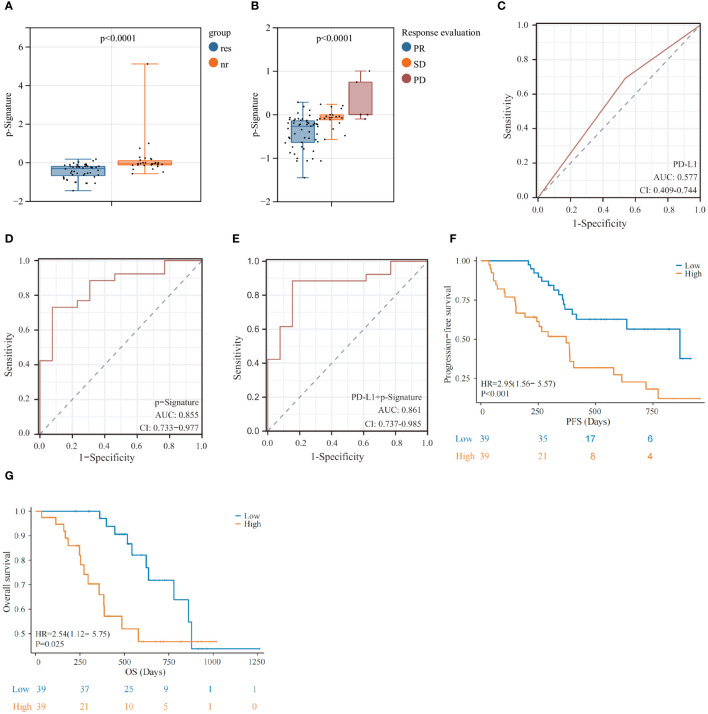
Prognostic predictive performance of the exLR-based p-Signature. **(A)** p-Signature between responders and non-responders in LUSC patients. **(B)** The patients with different best immunochemotherapy treatment response (partial response [PR], stable disease [SD] and progressive disease [PD]) demonstrated significant differences in p-Signature levels. **(C–E)** The ROC curve of PD-L1 **(C)**, p-Signature **(D)** and combined PD-L1 levels and p-Signature **(E)** in patients who received tissue PD-L1 detection. **(F, G)** Kaplan-Meier survival analysis (log-rank test) of progression-free survival (PFS) **(F)** and overall survival (OS) **(G)** among LUSC patients with different p-Signature levels.

Higher levels of p-Signature were observed in non-responders than those in responders (p<0.0001) (**Figure 4A**). The p-Signature aligns consistently with the best efficacy status assessed by imaging, with highest p-Signature levels in the PD groups and lowest levels in the PR group (**Figure 4B**). Furthermore, we conducted tumor PD-L1 assessment in 47 patients. The ROC analysis demonstrated that the AUC of tissue PD-L1 levels in distinguishing responders from non-responders is 0.577 (95% CI: 0.409-0.744) (**Figure 4C**). However, this accuracy is notably lower than using the p-Signature alone (AUC: 0.855, 95% CI: 0.733-0.977) (**Figure 4D**). When combining the PD-L1 status with the p-Signature, the AUC was 0.861 (95% CI: 0.737-0.985) (**Figure 4E**), which was similar to the AUC achieved with the p-Signature alone. This finding suggested that tumor PD-L1 level may not be an optimal predictive biomarker for immunochemotherapy. Then, we divided the patients into high and low-score group based on the median p-Signature value. Patients in the high-score group experienced significantly poorer PFS (p<0.001) (**Figure 4F**) and OS (p=0.025) (**Figure 4G**) than those in the low-score group, median progression-free survival (mPFS) was 868 days for low-score group vs. 372 days for high-score group, median overall survival (mOS) was 878 days for low-score group vs. 579 days for high-score group. Univariate analysis demonstrated that PD-L1 status and p-Signature were significantly associated with shorter PFS and OS(p<0.1), with the p-Signature acting as a promising predictor independent of other clinicopathological variables (**Table 2**). Collectively, our results suggested that the EV based p-Signature serves as valuable predictive factor, enhancing the predictive performance for clinical outcomes in LUSC patients receiving immunochemotherapy.

**Table 2:** Univariate and multivariate Cox analysis for PFS and OS in LUSC patients.

Variable	Categorization	Progression-Free Survival		Overall Survival	
		Univariate HR(95% CI)	Multivariate HR(95% CI)	Univariate HR(95% CI)	Multivariate HR(95% CI)
**Age**		1.025 (0.974 - 1.078), *P* = 0.352		1.041 (0.976 - 1.111), *P* = 0.218	
**Sex**	Male vs. Female	0.754 (0.101 - 5.649), *P*=0.783		0.392 (0.050 - 3.075), *P* = 0.373	
**Smoking history**	Previous/current vs. Never	0.745 (0.100 - 5.580), *P* = 0.775		2.297 (0.282 - 18.741), *P* = 0.437	
**Stage**	IV vs. III	1.147 (0.495 - 2.657), *P* = 0.749		2.934 (0.913 - 9.429), *P* = 0.071	3.286 (0.960 - 11.247) *P* = 0.058
**PD-L1 status**	PD-L1<1% vs. PD-L1≥1%	2.800 (1.181 - 6.639), *P* = 0.019	2.368 (0.973 - 5.762) *P* = 0.058	3.928 (1.334 - 11.567), *P* = 0.013	2.876 (0.896 - 9.234) *P* = 0.076
**p-Signature**		5.909 (2.171 - 16.083), *P* < 0.001	4.972 (1.851 - 13.352) *P* = 0.001	5.324 (1.609 - 17.621), *P* = 0.006	4.056 (1.236 - 13.315) *P* = 0.021

HR, Hazard ratio.

### 3.6 Potential mechanisms of p-signature in predicting LUSC patient’s prognosis

**Figure 5:**
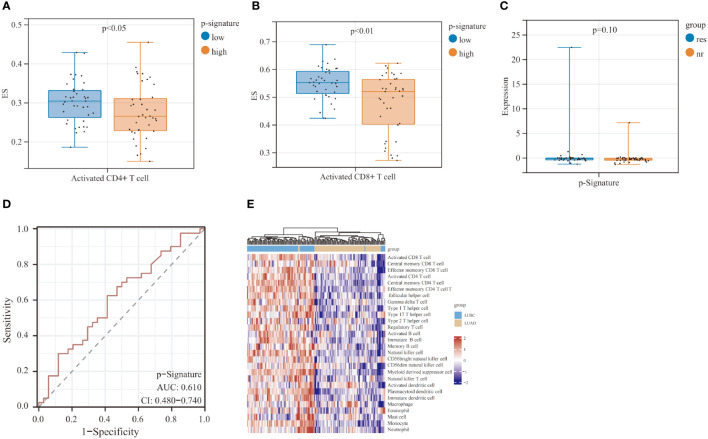
Prognostic predictive performance of p-Signature in the lung adenocarcinoma (LUAD) patients and the potential mechanism of p-Signature in predicting LUSC patient prognosis. **(A, B)** Activated CD4+ T cell **(A)** and activated CD8+ T cell **(B)** show significant difference between high and low p-Signature groups. **(C)** p-Signature between responders and non-responders in LUAD patients. **(D)** The ROC curve of p-Signature in responders and non-responders within the LUAD patients. **(E)** Heatmap of hierarchical clustering of cell types differentially observed in LUSC and LUAD patients.

To gain a deeper insight into the mechanism of the p-Signature in efficacy prediction, we utilized single sample gene set enrichment analysis (ssGSEA) to investigate the relative abundance of immune cell types in LUSC patients. The results revealed that the low-score group exhibited higher enrichment scores (ES) in activated CD4+ (**Figure 5A**) and CD8+ T cells (**Figure 5B**). To further explore the specificity of the p-Signature to LUSC patients, we applied the p-Signature to 74 LUAD patients. However, the p-Signature showed no difference between responders and non-responders in the LUAD patients (**Figure 5C**). The ROC analysis demonstrated that the AUC of the p-Signature in distinguishing responders from non-responders in LUAD is 0.610 (95% CI: 0.480-0.740) (**Figure 5D**). We also performed ssGSEA analysis in LUAD patients, and unsupervised hierarchical clustering unveiled a distinct separation of immune cell types between LUSC and LUAD patients (**Figure 5E**).

### 3.7 High level EV-derived CXCL8 correlates with reduced clinical benefit of immunochemotherapy

**Figure 6:**
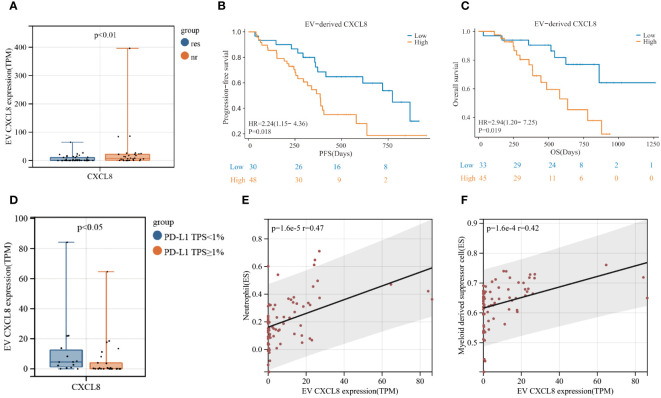
Elevated levels of EV-derived CXCL8 are associated with poor clinical efficacy of immunochemotherapy in LUSC patients. **(A)** Comparison of EV-derived CXCL8 expression levels between responders and non-responders. **(B, C)** Kaplan-Meier survival analysis (log-rank test) illustrating the impact of EV-derived CXCL8 expression on progression-free survival (PFS) **(B)** and overall survival (OS) **(C)** in LUSC patients. **(D)** Comparison of EV-derived CXCL8 expression levels between the PD-L1 TPS<1% group and PD-L1 TPS≥1% group. **(E, F)** Examination of the correlation between EV-derived CXCL8 levels and myeloid-derived suppressor cells (MDSC) **(E)** and neutrophils in LUSC patients.

EV-derived CXCL8 exhibited significant differences between responders and non-responders in patients with LUSC (**Figure 6A**). Moreover, we observed a notable extension in PFS (p=0.018) and OS (p=0.019) in the lower level EV-derived CXCL8 group (**Figures 6B, C**), mPFS was 773 days for lower level EV-derived CXCL8 group vs. 385 days for higher level group, mOS was 636 days for higher level EV-derived CXCL8 group, whereas lower level EV-derived CXCL8 group has not reached its mOS. Additionally, tissue PD-L1 TPS<1% group had a high level of EV-derived CXCL8 (**Figure 6D**). To further understand the role of CXCL8, we explored tissue-derived CXCL8 expression in the Cancer Genome Atlas (TCGA) - LUSC database. There was no significant difference between LUSC and noncancerous tissues in CXCL8 expression, and no survival benefit was observed (**Supplementary Figures S3A, B**). Furthermore, using ssGSEA, we discovered that the level of EV-derived CXCL8 has a positive correlation with myeloid-derived suppressor cells (MDSC) and neutrophils (**Figures 6E, F**).

## 4 Discussion

Our research contributes notably to the ongoing efforts to identify dependable biomarkers for immunotherapy-based treatments. To our knowledge, this study is pioneering in its analysis of the plasma exLRs transcriptome in advanced LUSC patients. Moreover, it’s the first to establish a predictive p-Signature that accurately forecasts the efficacy of first-line immunochemotherapy. This innovative approach, rooted in liquid biopsy techniques, offers a fresh and valuable perspective for pinpointing patients who are most likely to benefit from immunochemotherapy. By doing so, our study not only advances the field of precision medicine in oncology but also opens new pathways for personalized treatment strategies in lung cancer care.

In the quest for practical predictive biomarkers, new technologies that are easily implementable and capable of identifying novel biomarkers are in high demand. While PD-L1 expression level is the most widely used marker for immunotherapy efficacy prediction (26), recent evidence from the KEYNOTE 407 and RATIONALE 307 clinical trials demonstrated that advanced squamous lung cancer patients can benefit from Pembrolizumab plus chemotherapy regardless of their tissue PD-L1 status (5, 27). Furthermore, a retrospective analyze has shown that there was no predictive value of PD-L1 expression in squamous cell carcinoma in contrast to adenocarcinoma (6), suggesting that PD-L1 may not be an ideal biomarker for LUSC patients who received immunochemotherapy. Therefore, finding accessible biomarkers for patients with advanced squamous lung cancer is crucial. Liquid biopsy has emerged as a revolutionary strategy for cancer diagnosis and prognosis prediction (28, 29), given its noninvasive nature, ease of sample collection, ability to reflect the overall tumor status, and real-time monitoring capabilities. Previous studies have shown that plasma exLRs hold promise as biomarkers (30–33), and our previous research has indicated that CD160 has potential as a prognostic biomarker for LUAD patients receiving immunochemotherapy (19), albeit with limited success in LUSC prognosis prediction. Consequently, leveraging the availability of plasma samples and their capacity to reflect the systemic microenvironment, our study establishes a predictive signature based on plasma exLRs transcriptomics for patients with advanced/metastatic squamous lung cancer undergoing immunochemotherapy.

In our study, we first compared the plasma exLRs expression profiles of 78 patients with squamous lung cancer and 51 healthy subjects, revealing significant differences that suggest the potential utility of plasma exLRs as a biomarker. Subsequently, we evaluated the exLR profiles of responders and non-responders in 78 LUSC patients, and three exLRs were included in the construction of predictive signature of LUSC patients receiving immunochemotherapy. This signature efficiently distinguished responders from non-responders (respective cohort AUC = 0.904, prospective cohort AUC = 0.812). One of the key biomarkers identified, C-X-C motif chemokine ligand 8 (CXCL8/IL-8), is an angiogenic polypeptide expressed in multiple cancers (34). Previous studies have demonstrated that a decrease in serum IL-8 level is associated with decreased immunotherapy efficacy and longer OS in several advanced cancers (35–37). Moreover, a high level of CXCL8 is associated with decreased CD8+ T cell and neutrophil infiltration and contributes to immune evasion by regulating PD-L1 expression on macrophages (36, 38). Our study highlighted that EV-derived CXCL8 was correlated with a poor response to immunochemotherapy in LUSC patients, with high levels EV-derived CXCL8 being positively associated with MDSC and neutrophil infiltration. Additionally, the PD-L1 TPS<1% group exhibited high levels of EV-derived CXCL8. These findings underscore the potential of EV-derived CXCL8 as a biomarker for immunochemotherapy. The other two exLRs, succinate dehydrogenase complex assembly factor 1 (SDHAF1) and slingshot protein phosphatase 3 (SSH3), have not been extensively studied in the context of immunochemotherapy. SDHAF1 plays a crucial role in linking the citric acid cycle and electron transport chain (39, 40), while SSH3 has been identified as a potential antigen for developing mRNA-based vaccines against balder urothelial carcinoma (BLCA) and has been associated with colorectal cancer cell invasion and metastasis (41, 42). Our results reveal a positive correlation between these two exLRs and the response in LUSC patients who received immunochemotherapy, suggesting their potential roles that warrant further exploration. Overall, while our findings provide promising insights into the potential of these exLRs as biomarkers for immunochemotherapy response in LUSC patients, further studies are necessary to investigate their impact at the protein level and to elucidate the underlying mechanisms.

ExLRs are found in various bodily fluids due to active cellular secretion, and they can reflect the phenotype and functional states of their parent cells (43). Despite the strong predictive power of our p-Signature, ssGSEA analysis showed that a negative correlation between p-Signature and activated CD4+ and CD8+ T cells, suggesting that higher level p-Signature score may indicate an immune-evasion tumor immune microenvironment. Additionally, we observed that LUAD patients exhibited a significantly different TME compared to LUSC patients, and p-Signature was less effective in predicting prognosis among LUAD patients, highlighting its specificity for LUSC patients. However, the differences in TME between LUSC and LUAD require further investigation.

While our study has yielded valuable insights into the use of exLRs as biomarkers in LUSC treatment, we recognize certain limitations. Firstly, the patient cohort was relatively small and sourced exclusively from a single center, the FUSCC. Future studies should aim to include a more diverse and larger patient population from multiple centers, which would enhance the generalizability and robustness of our predictive model. Additionally, our study utilized an affinity-based method for EV isolation, which presents a limitation due to potential biases and variability affecting EV purity. To improve the accuracy and reliability of EV characterization, future studies should consider adopting methods such as size-exclusion chromatography or ultracentrifugation, as recommended by the Minimum Information for Studies of Extracellular Vesicles (MISEV) 2023 guidelines (44). These approaches are expected to provide a more representative EV population and further validate the predictive signature identified in this study. Furthermore, the specific roles of the three identified exLRs in modifying the TME warrant more detailed investigation. This is particularly true for EV-derived CXCL8, whose impact on the TME could provide deeper understanding and potentially influence future therapeutic strategies. Such expanded research efforts would be crucial in further validating and refining our findings.

In conclusion, our study marks a significant progression in identifying reliable biomarkers for immunochemotherapy in advanced LUSC. By analyzing the EV transcriptomic profiles of 78 advanced LUSC patients across both retrospective and prospective cohorts, we have successfully identified a LUSC-specific predictive signature (p-Signature) based on three exLRs. This p-Signature shows considerable potential in enhancing the precision of immunochemotherapy for lung cancer, potentially guiding more targeted and effective treatment strategies. Our findings represent a meaningful step forward in the pursuit of personalized medicine in the field of oncology.

## Data availability statement

The datasets presented in this study can be found in The Genome Sequence Archive, and accession number for RNA-seq data is PRJNA1132376.

## Ethics statement

The studies involving humans were approved by Fudan University Shanghai Cancer Center Medical Ethics Committee. The studies were conducted in accordance with the local legislation and institutional requirements. The participants provided their written informed consent to participate in this study.

## Author contributions

JW: Conceptualization, Funding acquisition, Investigation, Project administration, Supervision, Validation, Writing – review & editing. ZH: Conceptualization, Investigation, Project administration, Supervision, Writing – review & editing. XZ: Conceptualization, Data curation, Formal analysis, Methodology, Software, Validation, Visualization, Writing – original draft. JL: Data curation, Investigation, Methodology, Visualization, Writing – original draft. WY: Data curation, Formal analysis, Writing – review & editing. QL: Methodology, Resources, Software, Writing – review & editing. ZW: Conceptualization, Investigation, Writing – review & editing. HY: Data curation, Resources, Writing – review & editing. XW: Data curation, Resources, Writing – review & editing. HW: Data curation, Resources, Writing – review & editing. SS: Data curation, Resources, Writing – review & editing. XMZ: Data curation, Resources, Writing – review & editing.

## Glossary

**Table:**

ICIs	Immune Checkpoint Inhibitors
LUSC	Lung Squamous Cell Carcinoma
ExLRs	Plasma extracellular vesicle-derived long RNAs
NSCLC	Non-small cell lung cancer
PD-1	Programmed cell death 1
TPS	Tumor proportion score
TMB	Tumor mutation burden
MSI	Microsatellite instability
dMMR	Mismatch repair deficiency
EVs	Extracellular vesicles
LUAD	Lung adenocarcinoma
FUSCC	Fudan University Shanghai Cancer Center
ECOG	Eastern Cooperative Oncology Group
AJCC	American Joint Committee on Cancer
RECIST	Response Evaluation Criteria in Solid Tumors
PFS	Progression-free-survival
OS	Overall survival
ORR	objective response rate
CR	Complete response
PR	Partial response
SD	Stable disease
PD	progressed disease
TEM	Transmission electron microscopy
TPM	transcripts per kilobase million
FC	Fold change
FDR	False discovery rate
DEGs	Differentially expressed genes
GO	Gene Ontology
BP	Biological process
KEGG	Kyoto Encyclopedia of Genes and Genomes
LASSO	Least absolute shrinkage and selection operator
AIC	Akaike information criterion
PLR	Peripheral platelet to lymphocyte ratio
LMR	Lymphocyte to monocyte ratio
NLR	Neutrophil to lymphocyte ratio
AUC	Area under the curve (AUC)
ssGSEA	Single sample gene set enrichment analysis
ES	Enrichment scores
MDSC	Myeloid-derived suppressor cells
CXCL8	C-X-C motif chemokine ligand 8
BLCA	Balder urothelial carcinoma
