# Supplementary material for: Plasma extracellular vesicle long RNA profiling identifies a predictive signature for immunochemotherapy efficacy in lung squamous cell carcinoma
Source: Front Immunol. 2024 Aug 5;15:1421604. doi: 10.3389/fimmu.2024.1421604 (PMC11331801; doi:10.3389/fimmu.2024.1421604)
Supplement: Supplementary file 1 [file DataSheet_1.docx]

**Supplemental Methods**

**1. Plasma sample collection**

Blood samples were collected from all participants at baseline using 10 mL EDTA-coated Vacutainer tubes. The collected blood samples were centrifuged at 800 × g(~3000rpm) for 10 minutes at room temperature (25℃) using the human lymphocyte separation tube (Dakewe, Shenzhen, China) within 2 hours of collection. Then 1mL separated plasma was collected 1ml into an EP tube and further centrifuged at 16,000 × g (~13,000 rpm) for 10 minutes at 4℃ to remove debris. The resulting supernatants were aliquoted and stored at -80℃ until further use.

1. **EV purification**

EVs were purified using exoRNeasy Serum/Plasma Kit (Qiagen, Hilden, Germany) according to the manufacturer's protocol. 1. Add 1 volume buffer XBP to 1 volume of plasma. Mix well immediately by gently inverting the tube 5 times. 2. Add the sample/XBP mix onto the exoEasy spin column and spin the device for 1 min at 500 x g. Discard the flow-through and place the column back into the same collection tube. 3. Add 3.5 ml XWP (For Maxi kit add 10 ml) and spin 5 min at 5000 x g to wash the column and remove residual buffer. Discard the flow-through together with the collection tube. 4. For RNA isolation, the EVs were lysed with QIAzol. For EM and WB analysis, the EVs were eluted with 400ul XE elution buffer (Qiagen, Cat. No. 76214). Ultrafiltration may be used to reduce eluate volume and exchange buffer conditions with PBS using Amicon Ultra-0.5 Centrifugal Filter 10kDa (Merck Millipore, Germany).

1. **ExLR isolation**

We performed RNA isolation as previously described (Clinical Chemistry 2019, PMID: 30914410). We used miRNeasy (RNeasy MinElute spin column) included in exoRNeasy Serum/Plasma Kit to isolate total EV RNAs. EVs were lysed on the column with a 5-minute incubation in QIAzol (Qiagen, USA) at room temperature (15–25°C). 90 µl chloroform was then added to the QIAzol eluate, followed by centrifugation for 15 minutes at 12,000 × g at 4 °C. The upper aqueous phase was transferred to a new collection tube and mixed with 2 volumes of 100% ethanol thoroughly. 700 µl sample was then transferred into the RNeasy MinElute spin column (Qiagen, USA), followed by centrifugation at ≥8000 x g (≥10,000 rpm) for 15 s at room temperature (15–25°C) to discard the flow-through. After washing sequentially with buffer RWT and buffer RPE, the EV RNAs were eluted using RNase-free water. The EV RNAs were immediately subject to RNA sequencing (RNA-seq) library preparation. The remaining EV RNAs were stored at -80 °C for use within two weeks if necessary. To minimize potential bias, the sample handling staff were blind to the clinical information of the samples in the whole process.

1. **Transmission Electron Microscopy (TEM)**

The EVs were diluted with PBS and then concentrated through ultrafiltration using Amicon Ultra-0.5 Centrifugal Filter 10kDa (Merck Millipore, Germany). Negative staining with phospho-tungstic acid was employed to identify the EVs. Fifty microliters of resuspended EVs were placed on a parafilm membrane. A copper mesh with a formvar supporting membrane was covered with the EV suspension and allowed to float for 3-10 minutes to allow sample absorption into the supporting membrane. Subsequently, 50 µL of 2% phosphotungstic acid was applied onto the parafilm membrane. The excess fluid was absorbed from the edges of the copper mesh using filter paper. The copper mesh, which absorbed the sample, was then covered with 2% phospho-tungstic acid and floated for 3 minutes. After the staining solution was absorbed with filter paper, the sample was dried for 10 minutes under incandescent light. Transmission electron micrographs were obtained using a transmission electron microscope (Philips CM120, Tokyo, Japan) operating at a voltage of 120kv.

1. **Size Distribution Measurement**

The size distribution analysis of the EVs was conducted using a Flow NanoAnalyzer (NanoFCM Inc., U30E, Xiamen, China) following the manufacturer’s instructions. To establish a calibration curve correlating particle size with side scattering intensified, a set of monodisperse silica nanoparticles was synthesized and utilized as size reference standards. Then, the side scattering (SSC) distribution histogram of the mixture was obtained. The SSC intensity of every vesicle was converted into its corresponding vesicle size. One hundred mL phosphate-buffered saline (PBS) resuspended EV samples and 100 mL PBS (blank control) were analyzed using the same instrument settings. Finally, By applying calibration curve, the size of each vesicle was determined.

**4. Western Blot Analysis**

Peripheral blood mononuclear cells (PBMCs) were isolated using the human lymphocyte separation tube (Dakewe, Shenzhen, China) following the manufacturer’s instruction. PBMCs and the concentrated EVs were lysed in RIPA buffer (1% NP40, 0.5% deoxycholate, 0.1% sodium dodecyl sulfate [SDS] in Tris-buffered saline) containing complete protease inhibitors on ice for 30 minutes. The lysates were then subjected to protein quantification. Equal amounts of protein from EVs and PBMCs were loaded onto 10% SDS-polyacrylamide gels and subsequently transferred to nitrocellulose membranes (Bio-Rad, Hercules, CA, USA). The membranes were probed with the following antibodies: anti-CD63 (to be specified), anti-TSG101(to be specified) , and anti-Calnexin(to be specified). Detection was performed using peroxidase-conjugated secondary antibodies (to be specified) and visualized using the ECL SuperSignal West Pico substrate (Thermo,USA)
