# Supplementary material for: Plasma extracellular vesicle long RNA profiling identifies a predictive signature for immunochemotherapy efficacy in lung squamous cell carcinoma
Source: Front Immunol. 2024 Aug 5;15:1421604. doi: 10.3389/fimmu.2024.1421604 (PMC11331801; doi:10.3389/fimmu.2024.1421604)
Supplement: Supplementary file 2 [file Image_1.pdf]

Supplemental Figure 1

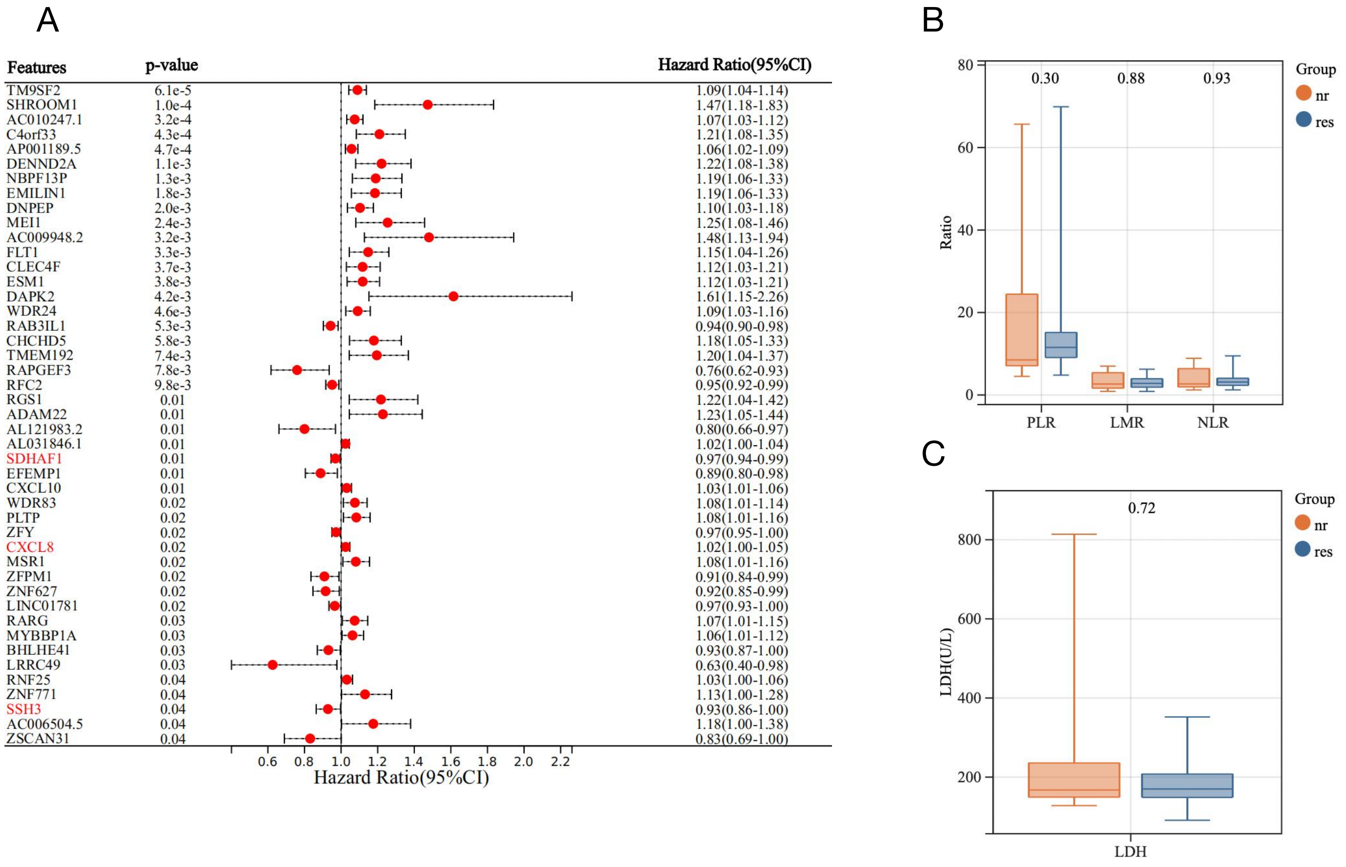

Supplemental figure1. Comparison of responders and non-responders in the retrospective cohort of Lung Squamous Cell Carcinoma (LUSC) patients. (A) Univariate Cox proportional hazards regression analysis of 45 prognostic-related genes with PFS. (B) Boxplot showing that platelet to lymphocyte ratio (PLR), lymphocyte to monocyte ratio (LMR), neutrophil to lymphocyte ratio (NLR) (B) and serum LDH level (C) between responders and non-responders.

Supplemental Figure 2

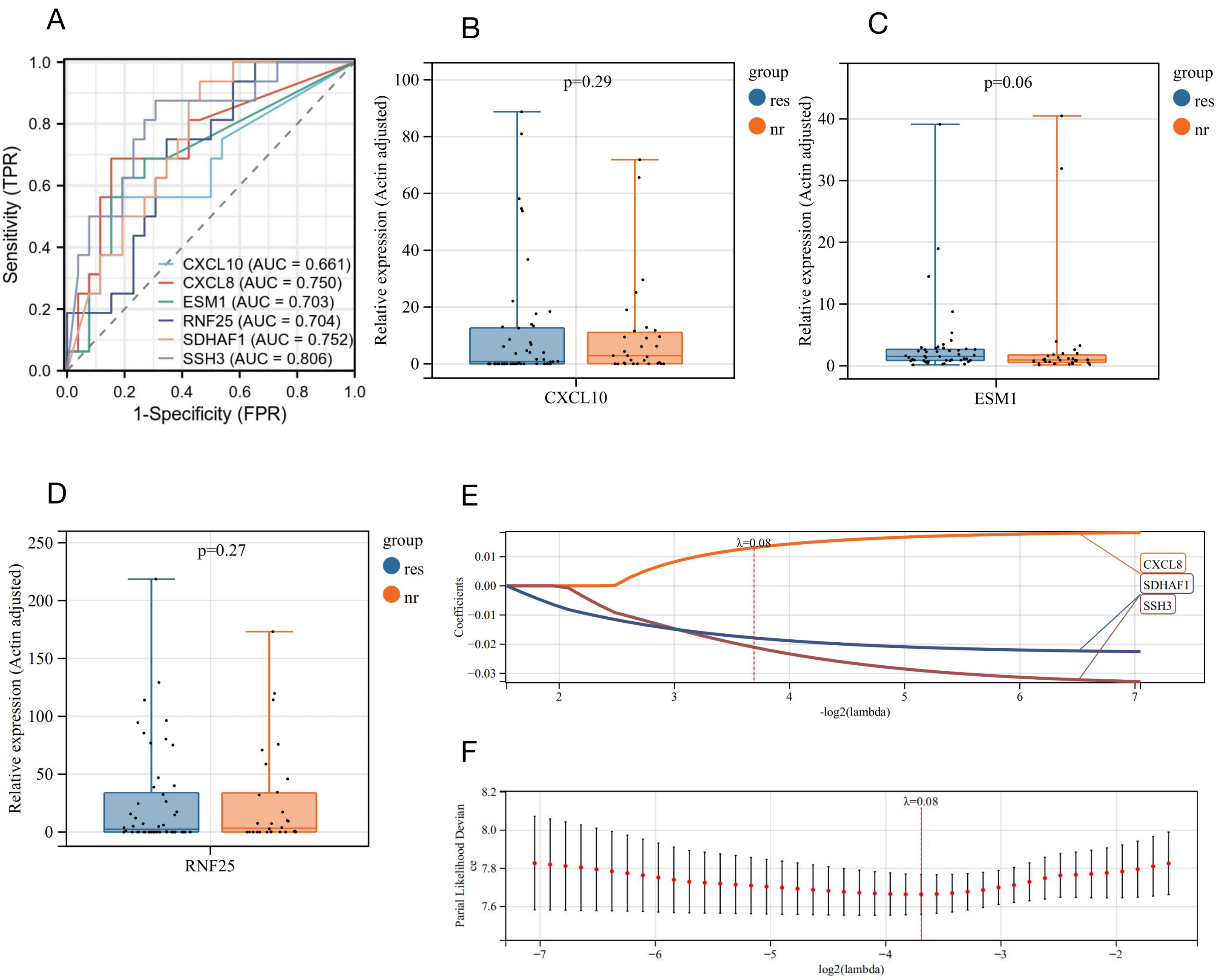

Supplemental Figure2. Construction of immunochemotherapy outcome predictive model. (A) ROC analysis of 6 candidate exLRs. (B, C, D) Relative RNA expression levels of 3 exLRs that did not show significant differences between responders and non-responders. (E, F) Construction of the predictive model utilizing the least absolute shrinkage and selection operator (LASSO) Cox regression algorithm.

Supplemental Figure 3

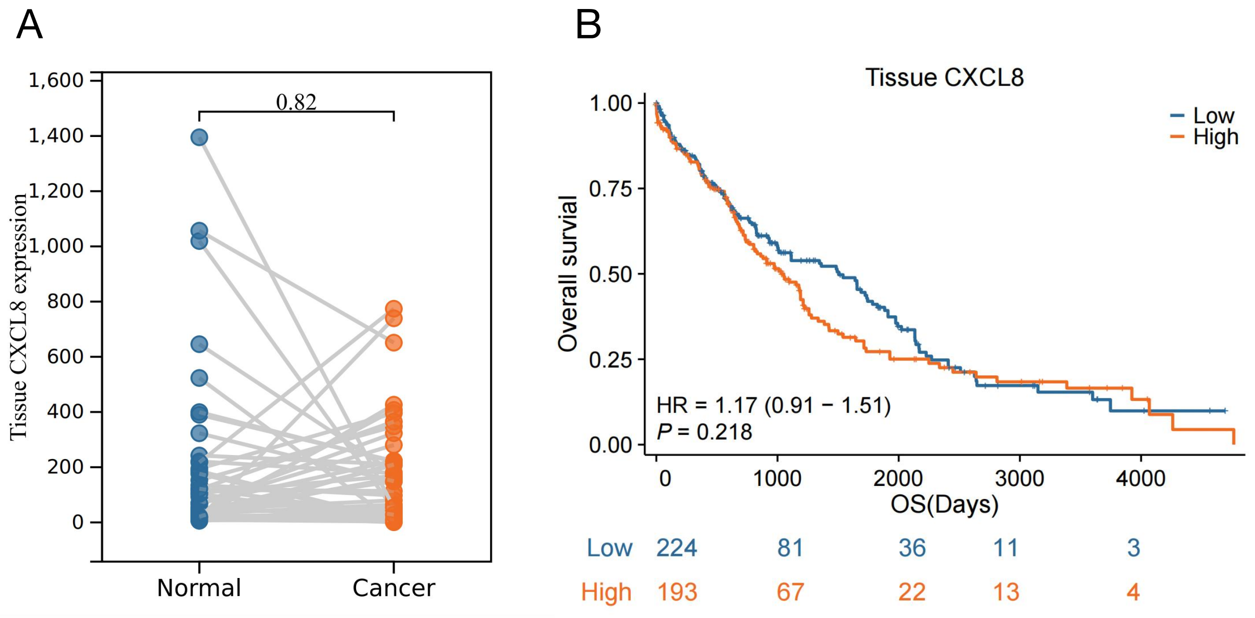

Supplemental Figure3. Prognostic predictive performance of Tumor-derived CXCL8. (A) Tumor-derived CXCL8 demostrated no significant difference with noncancerous tissues. (B) Kaplan-Meier survival analysis (log-rank test) of overall survival (OS) among TCGA-LUSC patients.
