# Supplementary material for: Plasma extracellular vesicle long RNA profiling identifies a predictive signature for immunochemotherapy efficacy in lung squamous cell carcinoma
Source: Front Immunol. 2024 Aug 5;15:1421604. doi: 10.3389/fimmu.2024.1421604 (PMC11331801; doi:10.3389/fimmu.2024.1421604)
Supplement: Supplementary file 3 [file Table_1.docx]

**Supplementary Table 1. The primers for Quantitative PCR (qPCR)**

|  | **Forward primer(5‘-3’)** | **Reverse primer(5‘-3’)** |
| --- | --- | --- |
| **RNF25** | TCCACTGCCACTGCCTTGCTCG | TTCCTGTTCCTGCTCCTGTC |
| **CXCL8** | CAGTGAAACTTCAAGCAAATC | AATCTTGTATTGCATCTGGC |
| **SDHAF1** | TTGCCAGCCTGACGACGGCG | CCGTCCGTCGGGGCGGGTCTC |
| **CXCL10** | ACTGCCATTCTGATTTGCTGC | ATGCAGGTACAGCGTACAGT |
| **ESM1** | GGTAATCTTACGTGGAGACAGGT | TTTGACTCACTGCGGTCTTCA |
| **SSH3** | TGCCTCAATGAGTGGACGGC | *CTGCTCCTGTTCTGAGGAC* |
| **Actin** | TTGTTACAGGAAGTCCCTTGCC | *ATGCTATCACCTCCCCTGTGTG* |

**Supplementary Table 2. General characteristics of the healthy and LUSC participants**

| **Characteristic** | **LUSC**  **(N=78)** | **Healthy**  **(N=51)** | **P value** |
| --- | --- | --- | --- |
| **Age, mean ± SD** | 65.47 ± 7.40 | 63.51±12.81 | 0.272 |
| **Gender, n (%)** |  |  | 0.263 |
| **Female** | 3 (3.8%) | 5 (9.8%) |  |
| **Male** | 75 (96.2%) | 46 (90.2%) |  |

**SD: standard deviation.**
